# 

**Published:** 1887-06

**Authors:** 


					﻿■Vnl. VIII.
JUNE, 1887.
No. 6.
THE
ntotiitttnipramiiontr:
> MOHTHIX
DEVOTED TO
DENTAL AND ORAL SCIENCE.
W. C. BARRETT, M. D., D. D. S.,
EDITOR.
The New York Dental Journal Association,
PUBLISHERS,
No. 35 West 46th Street, New York.
AND
TNo. aos Franklin St., UnfFalo, TN. Y.
$2.50 Per Annum in Advance,
Single Copies, 25 Cents.
Entered at the Post-Office, Buffalo, N. Y., as Second-Class matter.
				

